# Bridging the gap between systems biology and synthetic biology

**DOI:** 10.3389/fmicb.2013.00211

**Published:** 2013-07-25

**Authors:** Di Liu, Allison Hoynes-O’Connor, Fuzhong Zhang

**Affiliations:** Department of Energy, Environmental and Chemical Engineering, Washington UniversitySt. Louis, MO, USA

**Keywords:** systems biology, synthetic biology, microbial engineering, metabolic engineering, cell factory

## Abstract

Systems biology is an inter-disciplinary science that studies the complex interactions and the collective behavior of a cell or an organism. Synthetic biology, as a technological subject, combines biological science and engineering, allowing the design and manipulation of a system for certain applications. Both systems and synthetic biology have played important roles in the recent development of microbial platforms for energy, materials, and environmental applications. More importantly, systems biology provides the knowledge necessary for the development of synthetic biology tools, which in turn facilitates the manipulation and understanding of complex biological systems. Thus, the combination of systems and synthetic biology has huge potential for studying and engineering microbes, especially to perform advanced tasks, such as producing biofuels. Although there have been very few studies in integrating systems and synthetic biology, existing examples have demonstrated great power in extending microbiological capabilities. This review focuses on recent efforts in microbiological genomics, transcriptomics, proteomics, and metabolomics, aiming to fill the gap between systems and synthetic biology.

## INTRODUCTION

Recent advances in genetics and molecular biology, including genome sequencing, bioinformatics, and high-throughput experimentation, have enabled the collection of large sets of data. These data provide a comprehensive understanding of complex biological systems, boosting the development of both systems biology and synthetic biology.

Systems biology aims to develop novel methodologies to study the functionality of the biological system as a whole. When studying microorganisms, these methodologies not only help to understand how microbes adapt, evolve, and interact with other organisms (Navid et al., 2009; Zaneveld et al., 2011), but also reveal the profile and the dynamics of RNAs, proteins, and metabolites, elucidate their intracellular interactions, and uncover complex regulatory networks. Several review articles have discussed the role of systems biology in the study of microbes (Brul et al., 2008; de Lorenzo and Galperin, 2009; Heinemann and Sauer, 2010; Kohlstedt et al., 2010).

Synthetic biology focuses on constructing artificial tools to achieve particular functions. Microbes are excellent hosts for many important applications such as bioremediation, biodegradation, bioconversion, and bioproduction. Particularly, engineered microbes have been extensively used to produce therapeutic proteins, industrial enzymes, small molecular pharmaceuticals, chemicals, biofuels, and materials. Review articles focusing on engineering microbes as cell factories are also available (Picataggio, 2009; Gowen and Fong, 2011).

Although systems biology and synthetic biology focus, respectively, on science and technology, knowledge of systems biology guides the design of better synthetic biology tools, which can in turn provide insights to systems biology. Here we review the recent development of systems and synthetic biology methodologies for the understanding and control of genomics, transcriptomics, proteomics, and metabolomics in microbes. We also discuss the further possibilities for these two fields to benefit from each other.

## GENOMICS

As the basic unit of heredity, a gene is transcribed to an mRNA, which is translated to a protein, and all these molecules work together to perform complex functions within a living organism. Systems biology methodologies, such as whole genome sequencing, enable better understanding of gene function, which in turn allows the development of synthetic biology tools to manipulate genetics.

At the systems biology level, deciphering genetic codes provides valuable information on the structure and function of genes. Initiated in the 1970s, sequencing techniques have gone through striking development, and now fully automated DNA sequencing instruments coupled with high-throughput capabilities are available to many research labs. On average, bacterial genome sequencing can now be completed within hours or days, at the cost of $25 per Mb assembled sequence (Didelot et al., 2012; Loman et al., 2012). The analysis of the raw sequencing data is largely aided by bioinformatics, which integrates techniques from different disciplines, such as computer science and mathematics, to interpret biological data. Bioinformatics not only helps in genome annotation, but also provides insights on the corresponding protein functions and homologies between species. Proteogenomics, for example, unifies genomic data and protein identification techniques, allowing for new gene discovery and accurate gene annotation (Armengaud, 2010). Proteogenomics plays an important role in systems biology by providing a detailed picture of cell systems. For example, Banfield et al. (2005) implemented proteogenomics in the characterization of bacterial communities living deep in mine tunnels: specifically those that produce chemoautotrophic biofilms.

At the synthetic biology level, the ability to edit genetic sequences is the basis for manipulating any synthetic system, which creates an obvious need for such editing. Stimulated by this need, DNA synthesis methods have been developing fast over the past few years. Many methods have been developed in the pursuit of efficient, high fidelity, and low cost DNA synthesis techniques. For example, Tian et al. (2004) used photo-programmable microfluidic chips for multiplex gene synthesis, coupled with a hybridization-based method for error correction. The current cost for commercial gene synthesis is $0.28/bp or even lower (Genscript, Inc.), rendering genetic manipulation easier than ever. While chemical synthesis of the whole microbial genome has been demonstrated for *Mycoplasma mycoides*, at the current stage of development, complete chemical synthesis of microbial genome could be complicated and costly (Gibson et al., 2010). An alternative approach to construct large pieces of DNA or metabolic pathways is to assemble multiple existing DNA fragments. For example, the well-established Gibson DNA assembly approach utilized the activity of 5′ exonucleases to generate single-stranded DNA overhangs, which can then be sealed and ligated with high accuracy and efficiency using a DNA polymerase and a ligase (Gibson et al., 2009). This method allows scar-less assembly of multiple DNA fragments in one-pot. Other DNA assembly methods, such as CPEC (circular polymerase extension cloning; Quan and Tian, 2009) and Golden Gate (Engler et al., 2008), have their own advantages and are suitable for certain purposes (**Table 1**).

**Table 1:** Systems biology and synthetic biology tools and applications.

	Systems biology		Synthetic biology	
	Tools	Applications	Tools	Applications
Genomics	DNA sequencing	Gene identification, annotation, protein identification	Gibson, CPEC, etc.	Restriction enzyme-free DNA assembly
			Golden Gate	DNA assembly better for library construction
	Bioinformatics		MAGE, CAGE	Genome wide modification
	Proteogenomics		Gene cluster refactoring	Orthogonal genetic components
Transcriptomics	RNA microarray, RNA-Seq	Gene function interpretation, transcriptomic dynamics	Synthetic promoters, ribozymes, aptamers, sRNAs, etc.	Regulate transcript and translation
			RBS calculator	Control translational level
Proteomics	SRM	Protein detection and identification	Modular design of proteins	Build artificial proteins or regulate protein activities
			Post-translational modification	
			Computational protein design	
Metabolomics	GC–MS, LC–MS, NMR, etc.	Identification of novel metabolic pathways, bottleneck steps	Key enzyme overexpression, mutation, and deletion	Optimize metabolic pathways
	Computational tools, FBA, MFA, etc.		Global regulator engineering	
			Synthetic transporter	Control metabolite secretion

By the traditional homologous recombination, genomic DNA can be inserted, deleted, and mutated at one site each time (Datsenko and Wanner, 2000). Recent research advances have led to modification of many targeted locations in the chromosome simultaneously. Wang et al. (2009a) demonstrated multiplex automated genome engineering (MAGE), a method based on oligo-mediated allelic replacement, as an efficient tool to introduce mutations to the microbial chromosome, generating rich genome diversity with tuned properties. This technique was applied to optimize multiple genetic components in the 1-deoxy-D-xylulose-5-phosphate (DXP) biosynthesis pathway, and achieved a more than fivefold increase in lycopene production within 3 days. Furthermore, in order to improve the efficiency of MAGE, the conjugative assembly of genome engineering (CAGE) method was developed, which permits large-scale genome modification by assembling modified genome parts. Combining these two techniques, researchers successfully demonstrated the ability to replace all the TAG stop codons with TAA in *E. coli* (Isaacs et al., 2011). The engineered strain has a free TAG codon that could be used in areas such as the incorporation of unnatural amino acids.

Even with these powerful synthetic biology tools available, functional construction of synthetic systems is still hindered by the hidden and complex regulation in the host cell. This fact requires that genetic components be well characterized and mutually orthogonal. Researchers recently developed a systematic process for refactoring the nitrogen fixation gene cluster, in which they changed the codons of essential coding sequences and put them under the control of synthetic parts, separated by synthetic spacer sequences (Temme et al., 2012). By removing the native regulations, the underlying interactions are simplified, which facilitates the engineering of the nitrogen fixation system in a non-nitrogen-fixation host. All the above-mentioned synthetic biology approaches provide systems biologists new tools to understand gene functions and complex genetic regulatory networks.

## TRANSCRIPTOMICS

Involved in both transcription and translation, RNA molecules serve as the link between genes and proteins. While systematic analysis of transcript profiles reveals gene expression patterns, synthetic regulation of these transcript levels can alter protein concentrations. A systematic understanding of transcriptomics is essential for designing synthetic regulatory systems.

The information obtained from genetics can be more precisely understood if we take a step further to the transcriptional level. By quantifying the expression level of related genes under different conditions, an RNA microarray was developed to facilitate the interpretation of the genome function and regulation patterns. This method is high-throughput and inexpensive, but limitations do exist, including the requirement of genome sequence information and errors caused by cross-hybridization (Wang et al., 2009b). Another technique, RNA-Seq, overcame these limitations by enabling the direct sequencing of RNA transcripts. It allows precise detection and quantification of transcripts to a single-base resolution, and can be applied to species with unknown genome sequences. More importantly, it provides a powerful tool to understand the transcriptomic dynamics by monitoring gene expression levels (Mortazavi et al., 2008; Wilhelm et al., 2008). Although challenges regarding library construction and bioinformatics data analysis remain, this method revolutionizes the way scientists analyze transcriptome data (Wang et al., 2009b).

For any synthetic system to function properly, it is key to regulate the expression of the genes involved. Gene expression can be regulated at different stages, among which transcriptional regulation often plays the most important role (Lodish et al., 2000; Romanel et al., 2012). Synthetic control of transcript levels can be achieved by varying the transcription initiation rate, transcript stabilities, or transcription termination frequency. Control of transcription initiation rates by constitutive or inducible promoters with various strengths have been used for many years. This approach still serves as the most effective and robust method for static control of the transcript level. However, sometimes it is advantageous to regulate a gene dynamically according to the environments and the metabolic status of the cell (Holtz and Keasling, 2010). This becomes particularly important when improving the robustness of a synthetic biological system, where many parameters could change as environmental conditions vary. The knowledge of transcription-level regulation from systems biology has inspired synthetic biologists to utilize natural elements to build synthetic regulatory tools. A recent work demonstrated the construction of a dynamic sensor-regulator system, in which transcription initiation rate of several heterologous genes are dynamically controlled by the cellular concentration of a key metabolite (Zhang et al., 2012b). In detail, biosensors specific to fatty acyl-CoAs, key intermediates in the biodiesel biosynthetic pathway, were engineered to control both the biosynthesis and the consumption of acyl-CoAs. Similar to natural regulatory systems, the synthetic control tool optimized gene expression levels, preventing the production of unnecessary RNAs and proteins and improving the efficiency of the biodiesel pathway. As a result, biodiesel titers were increased by threefold, reaching 28% of the theoretical yield. Besides the regulation of transcription initiation, mRNA stability can be regulated as well; control of the mRNA degradation rate, folding rate, and ribozyme activity by small molecules could allow for dynamic control of transcript activities in response to metabolites (Beisel et al., 2008; Carothers et al., 2011; Michener et al., 2012). For example, Babiskin and Smolke (2011) constructed an RNA device based on an RNase III enzyme, where they coupled RNA aptamers to the Rnt1p hairpins. Binding to ligand induced a conformational change on the RNA, which inhibited the self-cleavage activity and stabilized the transcript. Furthermore, transcription termination has been regulated by small RNA molecules that interact with mRNAs (Lucks et al., 2011). The naturally occurring transcriptional control mechanism in the *Staphylococcus aureus* plasmid pT181 uses an antisense RNA to induce a conformational change that exposes a transcription termination site. By producing mutations in the attenuator sequence, researchers were able to create variants that responded to unique antisense RNA molecules. These orthogonal signals were used in tandem to construct logic gates and an RNA-mediated transcriptional cascade.

Once a gene has been transcribed, cellular protein levels can be tuned at the translational level by engineering the ribosome binding sites (RBSs). Salis et al. (2009) developed a mathematical model, called the RBS calculator, to compute the RBS strength. This model considers the energies involved in rRNA–mRNA interaction, mRNA folding, tRNA binding, and the energetic cost of sub-optimal spacing between the RBS and the start codon (Salis, 2011). This computational tool was proved effective in designing RBS sequences to control relative protein levels.

The above-mentioned methods allow the synthetic regulation of a single gene or of multiple genes at either the transcriptional or the translational level. They are particularly powerful when optimizing a specific metabolic pathway. However, to improve the overall behavior of engineered microbes, such as tolerance toward chemicals or stress conditions, it might be useful to regulate gene expression at the whole genome-scale (Zhang et al., 2012a; Woodruff et al., 2013). For example, Alper et al. (2006) employed a global transcription machinery engineering (gTME) approach to improve both ethanol tolerance and production of a yeast strain. In this study, two proteins that regulate the global transcriptome (SPT15, a TATA-binding protein, and TAF25, TATA-binding protein-associated factor) were subject to random mutagenesis via error-prone PCR. These mutant libraries were introduced to yeast and subject to screening for their abilities to grow in the presence of ethanol. Strains selected from this approach confer both enhanced ethanol tolerance and efficient glucose to ethanol conversion. This study serves a good example for using synthetic biology tools to modify microbes at the systems level.

## PROTEOMICS

Proteins are ubiquitous in biological systems; their complex structure allows them to perform innumerable functions, such as transport, catalysis, signaling, and regulation. Thus a systematic understanding of proteomics, including protein structure, function, concentration, and interactions with other molecules must precede the development of novel synthetic systems.

Experimental protein studies rely heavily on proteomic technologies and instrumentation. Selected reaction monitoring (SRM) is a powerful proteomic technique that can quantitatively detect small numbers of a specific protein. However, SRM can only be used to detect proteins for which assays have been developed. Previously, assay development was an arduous process, limiting the use of SRM (Doerr, 2010). Picotti et al. (2010) devised a high-throughput method for developing SRM assays that allowed them to analyze all the phosphatases and kinases in the proteome of *E. coli*. By synthesizing and analyzing libraries of synthetic peptides, 432 SRM assays were generated in less than 6 h of instrumentation time with an 89% success rate. The ease with which these assays can now be generated will enable a wide variety of new applications, potentially including whole-proteome analysis. For example, Singh et al. (2012) used SRM techniques to optimize flux within the mevalonate pathway in *E. coli*. Malmstroem et al. (2009) applied this technique to determine the average quantity of proteins per cell for 51% of the open reading frames (ORFs), or 83% of the proteome of *Leptospira interrogans*, a human pathogen. They verified their measurements using cryo-electron tomography performed on whole, individual cells, and concluded that the mass spectrometric technique could be quickly and efficiently applied to biological systems.

While systems biology provides information regarding the structure and function of natural proteins, synthetic biology, empowered with such knowledge, can lead to the design of proteins that perform novel functions in synthetic systems. One approach is to design proteins based on modularity (Nash, 2012). Proteins, as well as DNA, RNA, and other small molecules, can often be broken down into modules – discrete parts that perform a specific function. Modules may bind to ligands, transmit information, catalyze a reaction, or accomplish a myriad of other tasks. For example, there are binding domains that mediate protein–peptide interactions by recognizing certain peptide characteristics, such as the SH3 domain that recognizes proline-rich sequences and the PDZ domain that recognizes specific C-terminal sequences (Teyra et al., 2012). Based on the SH3 and PDZ domain, Dueber et al. (2009) constructed synthetic protein scaffolds, which recruit peptide-tagged enzymes for spatial organization of multiple enzymes from a metabolic pathway, preventing the diffusion of metabolic intermediates and improving overall pathway efficiencies. Similar designs have also allowed for the control of signal transduction pathways through synthetic protein–protein interactions (Good et al., 2011). Furthermore, protein function can also be regulated through post-translational modification. Wang et al. (2010) demonstrated that global protein acetylation allowed for quick responses to changes in environmental conditions, allowing cells to modify their metabolism based on the availability of various carbon sources. Researchers also characterized the role of regulatory enzymes involved in the reversible acetylation process, elucidating a potential global regulatory circuit. Such a system could be engineered for fast regulation of metabolism at protein levels. In addition, protein design using computational approaches has presented more engineering opportunities, providing artificial proteins with novel activities and specific interactions with nucleic acids, small ligands, and other proteins (Mandell and Kortemme, 2009).

## METABOLOMICS

Metabolomics focuses on the profile and dynamics of metabolites, revealing the activity of cellular enzymatic reactions as well as metabolic and catabolic pathways. Further, metabolic analyses can be used as diagnostic tools in the study of microbial cell status and environmental conditions. From the perspective of a synthetic biologist, engineering microbial metabolomics has direct links with applications: to degrade toxins (Dziga et al., 2012), herbicides (Sinha et al., 2010), and environmental pollutants (Chien et al., 2010), and to produce chemicals (Curran and Alper, 2012), pharmaceuticals (Paddon et al., 2013), and biofuels (Zhang et al., 2011; Peralta-Yahya et al., 2012).

Metabolite identification and quantification methodologies based on gas chromatography–mass spectrometry (GC–MS), liquid chromatography–mass spectrometry (LC–MS), and nuclear magnetic resonance (NMR) have been developed in recent decades to study metabolite profiles and dynamics (Ludwig and Viant, 2010; Shuman et al., 2011; Bueschl et al., 2013). Additionally, metabolic modeling tools, such as flux balance analysis (FBA; Curran et al., 2012) and metabolic flux analysis (MFA; Tang et al., 2009), were developed. While both FBA and MFA are based on stoichiometric calculation of metabolic reaction rates under pseudo steady state assumptions, MFA uses experimental data instead of targeting biological fitness functions, as does FBA. Further, the combination of metabolic analytical methodologies and modeling tools helps to characterize metabolic networks, to identify novel metabolic pathways and bottleneck steps, and to study the responses of metabolic flux toward genetic modifications or under various environmental conditions.

Guided by knowledge from systems biology, synthetic biology aims to engineer microbial metabolomics, directing the flow of metabolites to desirable pathways. This includes constructing novel biosynthetic pathways for the production of useful molecules and engineering biodegradation routes for environmental applications (Huang et al., 2006). The above-mentioned metabolite analysis methodologies provide diagnostic tools for synthetic pathways, improving their productivity. Various modeling techniques consider metabolic network interactions and predict the optimal genetic modifications for targeted chemical production (Segre et al., 2002; Burgard et al., 2003; Ranganathan et al., 2010; Yang et al., 2011). For example, Ranganathan et al. (2010) developed an OptForce procedure to identify the potential targets to improve fatty acid production. This method used the flux measurements in a wild type strain, and simulated the optimal flux changes that should be made in the engineered strain for optimal production. The flux changes are then used to identify promising genetic interventions. The consistency between the computational results and the experimental measurements suggested that cell metabolism could be directed through programing design (Ranganathan et al., 2012). Another method developed by Flowers et al. (2013) can systematically identify, instead of one enzyme at each time, multiple target enzymes, whose expression levels could be simultaneously manipulated to obtain the desired phenotype. This method improved the computation efficiency dramatically.

The metabolic profile and regulatory networks obtained from systems biology studies also inspired scientists to develop synthetic control tools at the global level. In a recent study, a global transcription factor that controls multiple genes involved in fatty acid biosynthesis, degradation, and membrane transport was engineered (Zhang et al., 2012a). Overexpression of this single regulatory protein caused global-scale metabolic changes and was able to increase fatty acid production by fivefold, more significant than the overexpression of many single enzymes in the fatty acid pathway (Zhang et al., 2012a). In addition, the transport of metabolites across the cell membrane can be controlled by pumps identified by systems biology or engineered through synthetic biology methodologies (Dunlop et al., 2011). These pumps are very useful to secrete the product, but not the intermediates, out of the engineered host cell, lowering the stress from chemical accumulation while simplifying downstream processes.

## CONCLUSION AND OUTLOOKS

The inherent complexity of genetics presents researchers in systems and synthetic biology with the formidable task of respectively understanding and manipulating natural genetic systems and their complex control elements. The current available tools and their applications in systems and synthetic biology are summarized in **Table 1**. In the coming years, advancements in genomics will lead to a further decrease in the cost of DNA synthesis, accelerating research. Transcriptomics will experience the development of a wide range of synthetic tools, including control through the modular use of synthetic promoters and RNA elements, e.g., untranslated region (UTRs), RBSs, antisense RNA, and ribozymes. Proteomics will provide a wealth of data in the form of proteome mapping, and metabolomics will incorporate these advances to achieve high yields of desired products.

**FIGURE 1:**
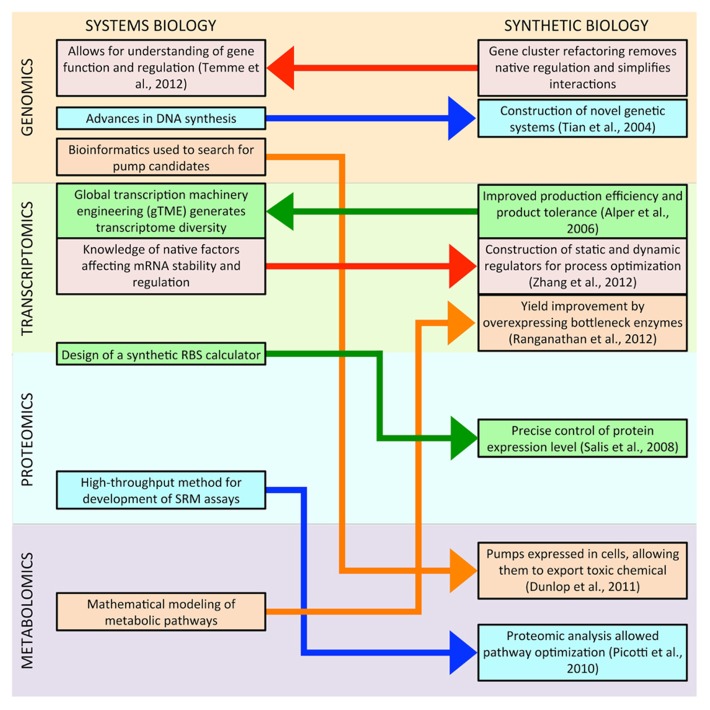
**This figure illustrates recent interactions between systems and synthetic biology.** In most cases, methodologies developed in systems biology have led to advances in synthetic biology. However, this trend may be changing, as research in synthetic biology has already begun to provide insight to systems biology, and there might be more research at the intersection of the two fields in the near future.

**Figure 1** illustrates the current interactions between systems and synthetic biology; synthetic biology is drawing more tools and knowledge from systems biology than it is reciprocating, but this trend is likely to change, and there might be more research at the intersection of the two fields in the near future. Deeper understanding of systems biology will provide more synthetic biology parts, such as biosensors that can be used for dynamic regulation of synthetic pathways (Zhang and Keasling, 2011). Systems biology knowledge will also make synthetic biology tools more reliable, enabling the precise control of transcription and translation regardless of the under-controlled gene (Mutalik et al., 2013). More powerful systems biology-based computational tools will simplify both the design and the optimization of synthetic metabolic pathways, improving titers and productivities (Colletti et al., 2011). Similarly, simplified genetic systems created in synthetic biology will provide systems biology with insight into the fundamentals of native gene regulation. Powerful synthetic quantification tools will allow the simultaneous collection of omic data at global scales in living cells. Overall, direct communication between systems and synthetic biologists regarding tools and knowledge will hasten progress in genomics, transcriptomics, proteomics, and metabolomics.

## Conflict of Interest Statement

The authors declare that the research was conducted in the absence of any commercial or financial relationships that could be construed as a potential conflict of interest.
